# Supplementary figures and images for: Functional genomics of chitin degradation by Vibrio parahaemolyticus reveals finely integrated metabolic contributions to support environmental fitness
Source: PLoS Genet. 2025 Mar 3;21(3):e1011370. doi: 10.1371/journal.pgen.1011370 (PMC11906056; doi:10.1371/journal.pgen.1011370)

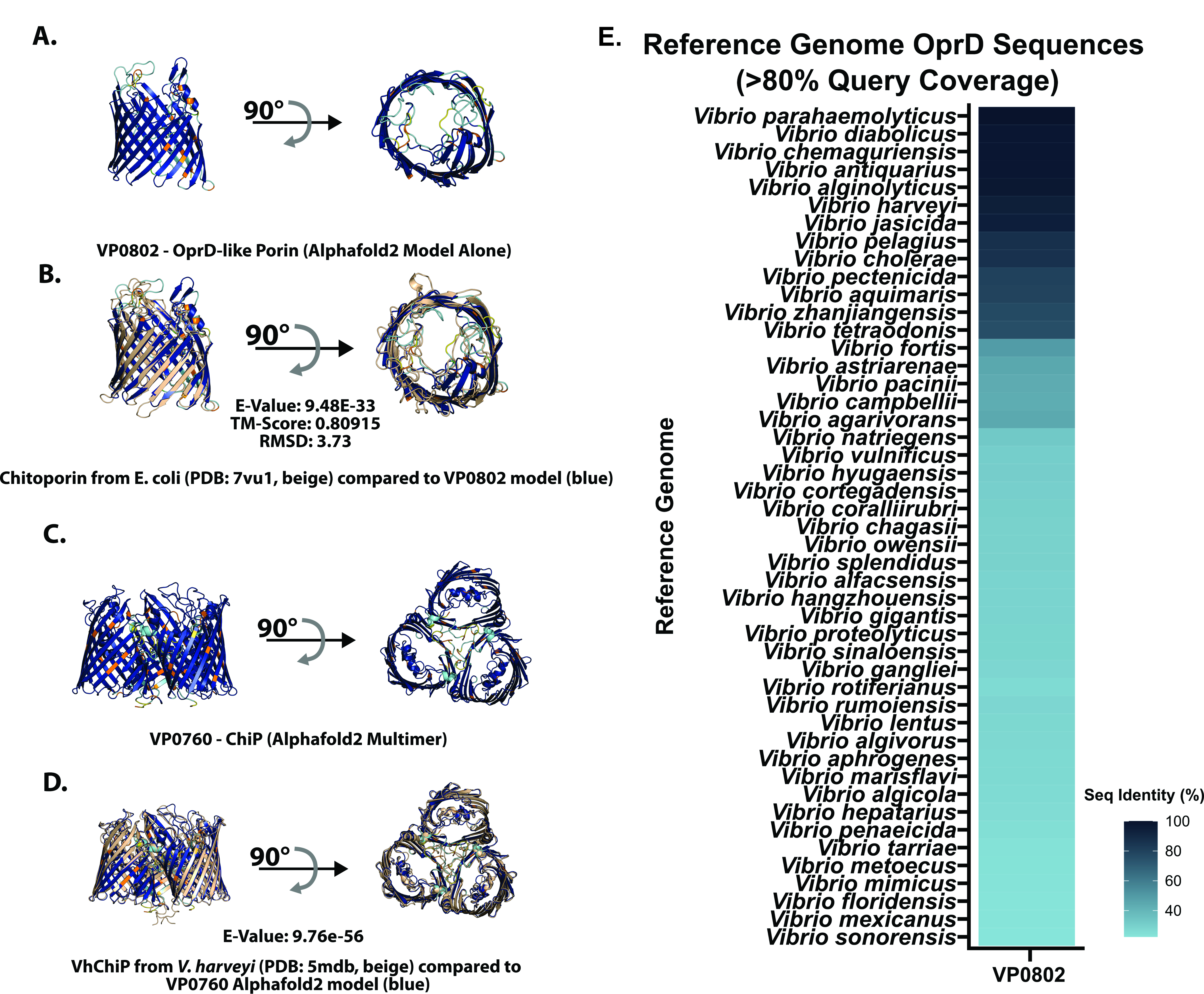

Supplement: S1 Fig — The analysis was performed with Reference Genomes for the indicated strains. (TIF) [file pgen.1011370.s005.tif]

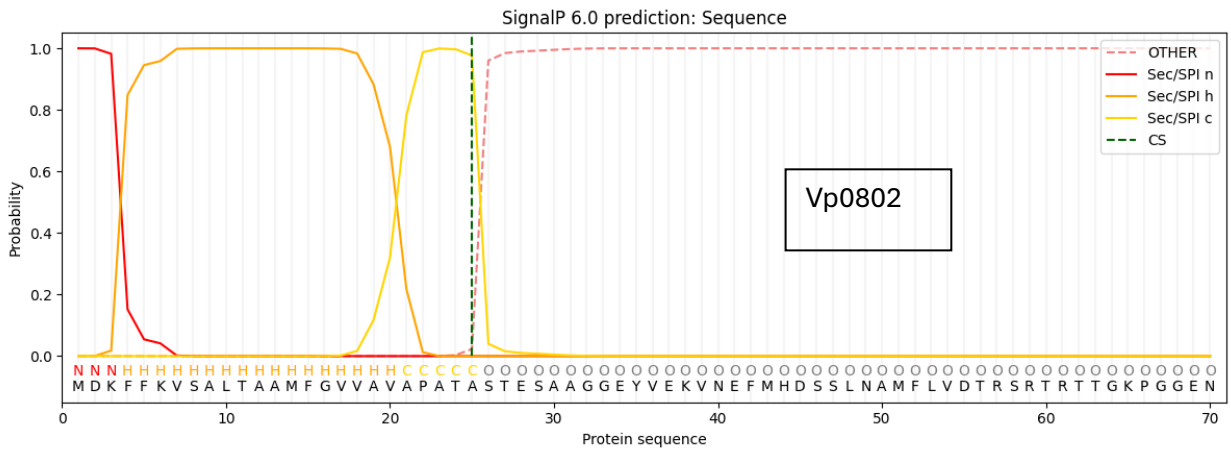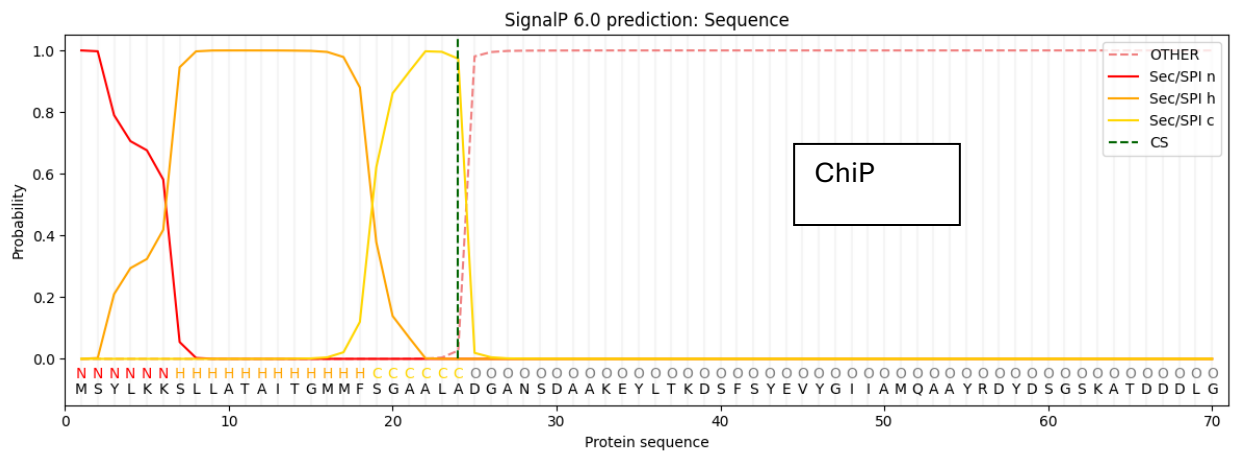

Supplement: S2 Fig — The leader sequence is located to the left of the dotted line with the initiating methionine as the first amino acid of the unprocessed pre-protein. (PDF) [file pgen.1011370.s006.pdf]

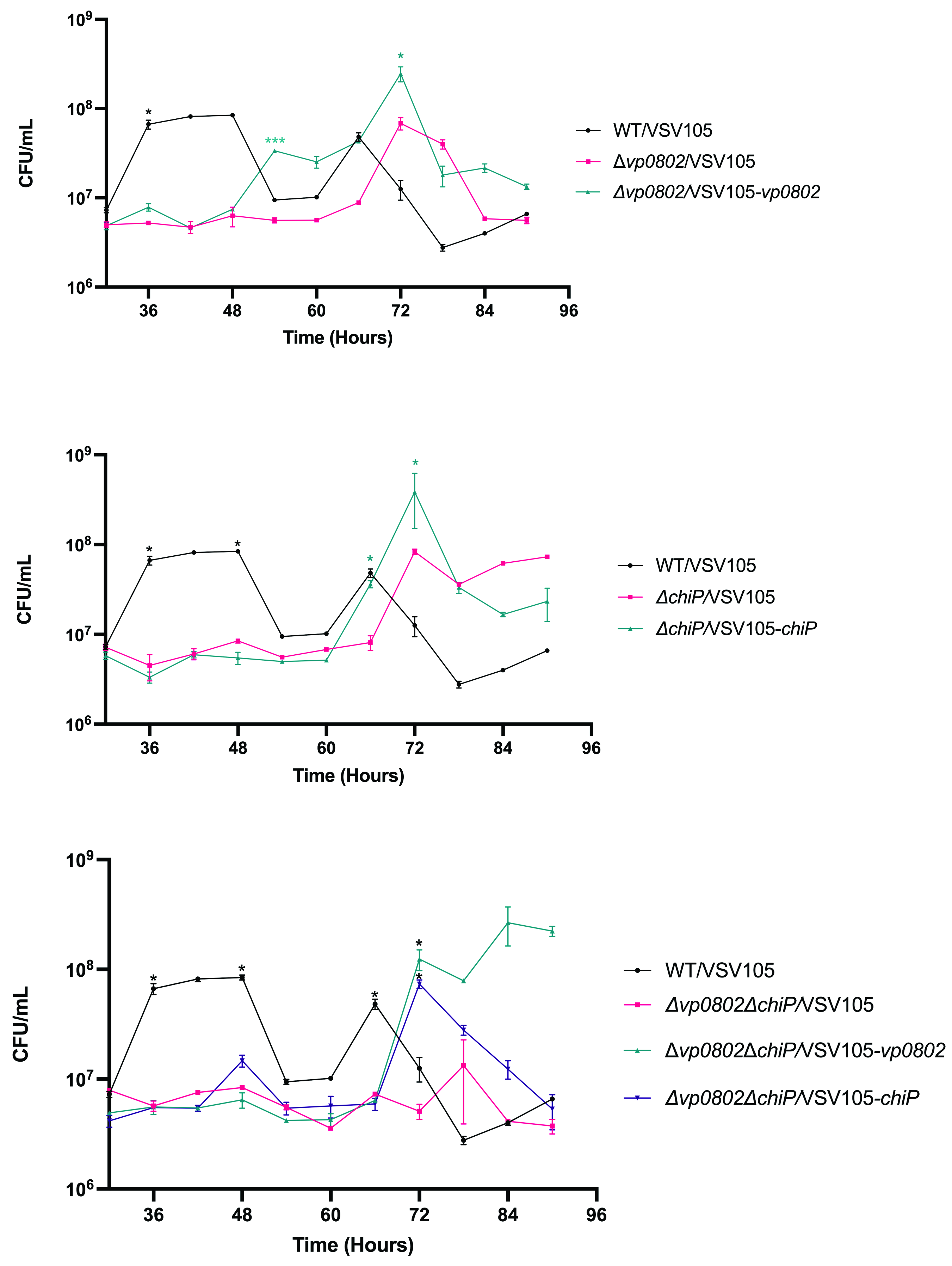

Supplement: S3 Fig — The indicated mutant strains (magenta) are compared to wild type (WT) V. parahaemolyticus harboring plasmid pVSV105 (black). The respective porin gene was cloned under control of its cognate promoter into pVSV105 and mobilized into the indicated bacterial strains. (TIF) [file pgen.1011370.s007.tif]

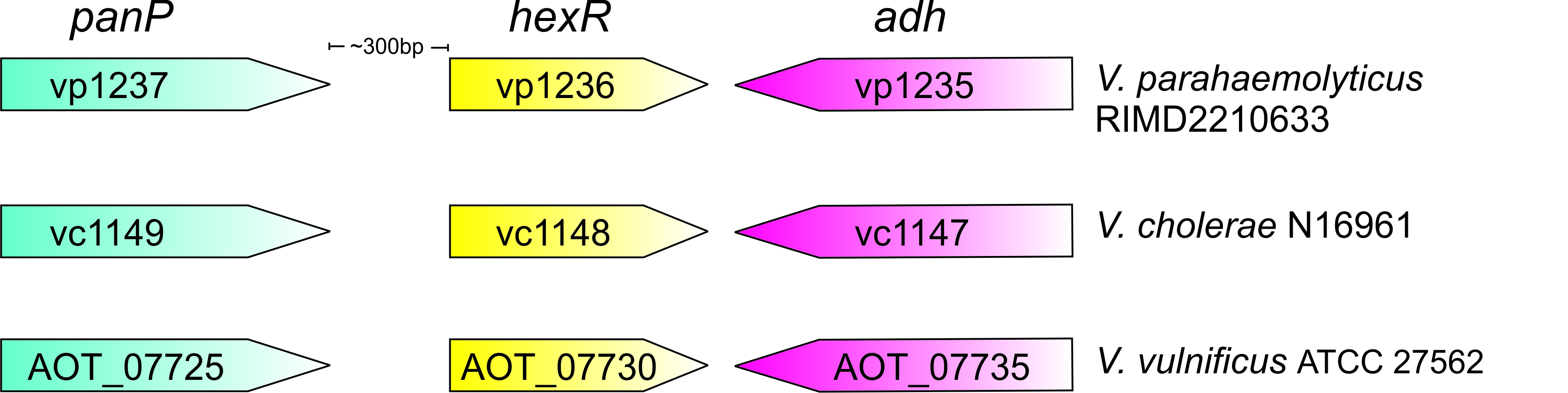

Supplement: S5 Fig — The teal and magenta colours for the hexR flanking genes indicate conserved panP and adh orthologs respectively. Note the presence of a ~300 bp intergenic region between panP and hexR which likely contains a gene promoter for hexR expression. The intergenic region was cloned with the hexR ORF (vp1236) and was found to successfully complement a hexR null mutant. (TIF) [file pgen.1011370.s009.tif]

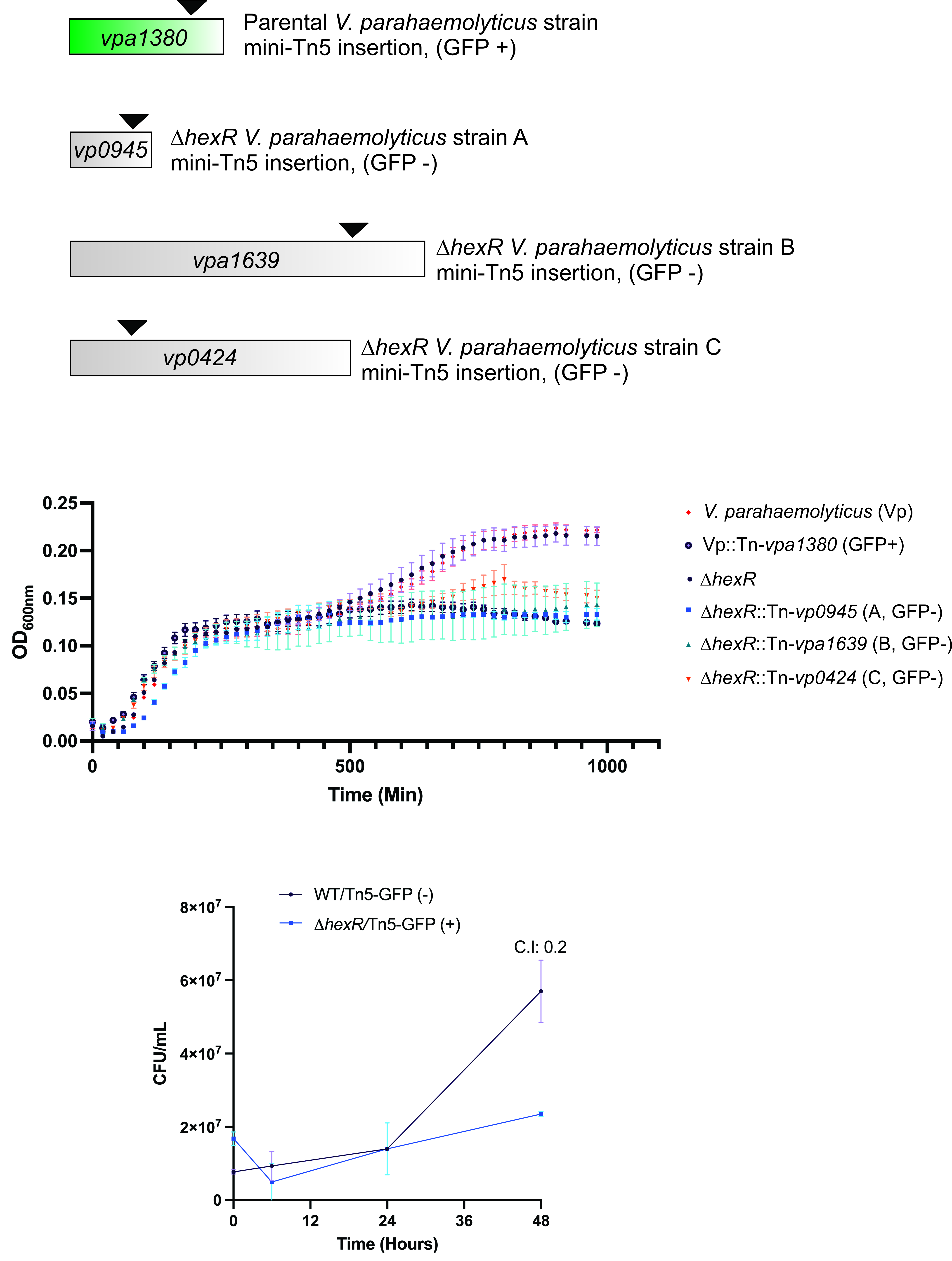

Supplement: S6 Fig — Top, Schematic showing the genomic location of a mini-Tn5 transposon insertion for the indicated strains. The wild type strain with an insertion in vpa1380 expresses a transposon-associated GFP allele using a chromosomal promoter, thus producing green fluorescence. The hexR mutant transposon insertions are non-fluorescent due to the absence of a suitable promoter position. Bottom, Growth curves in LB for the indicated bacterial strains. (TIF) [file pgen.1011370.s010.tif]
