# Supplementary material for: Functional genomics of chitin degradation by Vibrio parahaemolyticus reveals finely integrated metabolic contributions to support environmental fitness
Source: PLoS Genet. 2025 Mar 3;21(3):e1011370. doi: 10.1371/journal.pgen.1011370 (PMC11906056; doi:10.1371/journal.pgen.1011370)
Supplement: S4 Fig — Samples were derived from wild type bacteria (for Vp0802) (top) and from Δvp0802 (for ChiP) (bottom). Yellow highlighted sequences represent peptides accurately identified from the respective tryptic digests by mass spectrometry. In some cases, missed trypsin cleavages within peptides were observed. (PDF) [file pgen.1011370.s008.pdf]

>BAC59065.1 -Vp0802- [Vibrio parahaemolyticus RIMD 2210633]

MDKFFKVSALTAAMFGVVAVAPATASTESAAGGEYVEKVNEFMHDSSLNAMFLVDTRSRTRTTGKPGGEN

GDRWSRLNYSSYNAILDFTSGYHDGWVGADVAAYYSGDLYNDSLYNSNEGYLCNEISTCNNLDWGAGDGQ

QLKVYKAALK**FKANENFNAR**VGMLQAGGNGTIGNVWSFVPGTYR**GFELNAK**LGDFNLSYFGADQFTAPWL

LHEDDYAPALWSDTSWSYLHSLGLNGNLTDSLFFQVGLGQATGVKYANGIDWGAGQVTSYHNETDNTSYK

AYLKYTVSDRTTLAFDFYGVDDDVKYDGLGFHTGLSLNQSFGKLAWMSELR**YTDTDNRSDFVPR**TIHTYG

MNNGTWSQWWDALSDWNKAGELAWYNRLSYNQGNGWNYYLGFGYGSGADSAASGASGDWDYESEYAFNAT

VSYSLQSGALKGSTIR**LHGTILER**DEYAGAGDADETDLR**LQVLIPYSFH**

>BAC59023.1 -ChiP- chitoporin [Vibrio parahaemolyticus RIMD 2210633]

MSYLKKSLLATAITGMMFSGAALADGANSDAAKEYLTKDSFSYEVYGIIAMQAAYR**DYDSGSKATDDDLG**

GMQLNNESRIGFRGKKQFANFGPTFIWQIEGGYVDPSFGGEGAGLGERDTFVGFESASWGQVRLGRVLTP

MYELVDWPASNPGLGDVYDWGGAIGGAKYQDRQSNTIR**WDSPMYADK**FSLDIAAGAGDKAGLGAGDDYWG

GIAAHYK**LGPLQLDAAYEGNR**NIESEGQTWENNTYLVGVQGWFDNGISFFAQYK**YMEADASNGVNEK**QDA

MSAGLMYTTGDWQYKLGYAANFDLERDGKTINDTADDVLSAQVMYFVDPSAVLYVRAR**TLDFGDGASQLD**

KPAEARWKSADYDEFSVGVEYYF
