# Supplementary material for: Functional genomics of chitin degradation by Vibrio parahaemolyticus reveals finely integrated metabolic contributions to support environmental fitness
Source: PLoS Genet. 2025 Mar 3;21(3):e1011370. doi: 10.1371/journal.pgen.1011370 (PMC11906056; doi:10.1371/journal.pgen.1011370)
Supplement: S7 Fig — For the indicated genes, a putative HexR binding site (highlighted yellow) is shown in context of their respective predicted promoter elements. Promoter elements are shaded in grey with the -35/-10 elements underlined. (PDF) [file pgen.1011370.s011.pdf]

vp2732 (*pgi*, glucose-6-phosphate isomerase)

AGCCACGACTTCTTGCTTTTAATTGCAGTGTTTTTTGTGCCCACATACGTGTTCCTTTGGAAATTTATAAATTG
GCGTCTATCCTATTCCAATTAGAGCCTGTCATAAGTAAATGATATGATTCTTGCCATCATAAATGAATTGTTCAA
ATTCAGGGCAGGGAACGTGACCTAACTCAAGGTGAGTGTAAAAGCACTCGGTCATAATTGGCGCCTGAAA
AAAAATTACAAAGACTTGTTTTGTGGCAATTCGCCCAAACATCTAATTAATATTCTATTTGCTAAATCGGGGAT
TCCACAGTTTTCGCTAGACTGTAAGGAATGGAACCTACTGTAACATCGGGATAAATACCATGTTGAAAAAT

vpa1645 (glgX, glycogen debranching enzyme X)

ACACTTACTTTTTTCATTCTTAACTCCATTAAGGGTATTTATAATTCTGTCTTCCCTGCTCGCCCTCAGG
TGAATATAGGCAGTAAAAACGCACCTAAAGAAAGAGAGGTAAGATGGGAACGAATCCCCGTTTGTTTCAA
CAGTTATAAGACTACCCGAGGGGTACAAGGATGACTTCCTCCGCCCCAAACTAATTTAGGGGGGATGAGA
AGGGAGTAGAGAAATTACCGCATGGTGTTTTTTCGAGTCAGATCACCAATCTAGGGAGGAATTAGTAAGT
TTCTTCGGTTTTGAGCCGCTACAAAGTGATAGCTAATTCATATTTTTGACCCGAAGTCGTCATCTAAAGTAATT
AAATTACACGAATATTTTTCTATTTAAGGTGGTTGACCTTAGTAAGACAAAGTTTTTAACTTATAGTTTCCTAAAG
GTAGCAATAAATAATAAATAAAGTATCTCCGATGACACGACTTTTCTCTCGCCCCTACCCCCTAGGCGCAA
CGTTAAATAAAGAAGGCTGTAACTTCTCTATTCATGCTCCGGGTGTA

CONSENSUS (derived from RegPrecise, *Vibrio* genus)

TGTAAATTAAATTACA

TGAAAAA-AAATTACA- *pgi*
AGTAA-TTAAATTACA- *glgX*
