# Supplementary material for: Functional genomics of chitin degradation by Vibrio parahaemolyticus reveals finely integrated metabolic contributions to support environmental fitness
Source: PLoS Genet. 2025 Mar 3;21(3):e1011370. doi: 10.1371/journal.pgen.1011370 (PMC11906056; doi:10.1371/journal.pgen.1011370)
Supplement: Attachment — Submitted filename: Author Response R2.pdf [file pgen.1011370.s013.pdf]

Author Responses to reviewers' comments (in blue below)

Reviewer #1: Getz et al properly addressed my concerns in this revised manuscript. I am satisfied with the additional data and discussion. I am including below a few minor (cosmetic) points for the authors to consider. Congratulations on an interesting finding!

Minor comments:

Fig. 4B – the X-axis could start at t=0h to show the lag phase described in the text.
-This is now included.

Lines 181-2: not clear where one parenthesis starts.
-We have altered the sentence structure to improve readability.

Lines 182-4: is something missing in this sentence? It isn't clearly connecting with the preceding one (maybe the timing in which the described decline is apparent?).
-We agree and have altered the sentence structures here and before to improve readability.

Line 277: is it cell density or CFU?
We used cell density for initial growth comparisons between the parent and tagged GFP strains (S6 Fig). For the competition experiments, equivalent cell numbers were initially determined by optical density and validated by plate counts (CFU). During competition experiments, Viable plate counts (CFU/ml) were determined by differentiating between GFP+/- colonies at specific time points and plotted accordingly (Fig 6B).

Fig. 9A: it would be better to spell out the strains rather than to use numbers.

This has been done as recommended.

Fig. 9D and lines 364-6: I suggest caution in the interpretation of the swarming results. The differences between the repeats are quite dramatic (diameters range between ~45 and 85 mm for WT, for example). This is bound to affect statistical analysis and confidence.

We agree and have added statements in the results section to aid with interpretation and indicate some limitations of the experiment. See new lines 373-375.

Reviewer #2: This remains and interesting manuscript describing several genes not implicated prior in metabolism of chitin by Vibrio parahaemolyticus, which plays an important role in global carbon cycling. In this revised manuscript, the authors address most, but not all, comments by this reviewer.

Major comments
1. New lines 216-219. The authors do not adequately address the concern that naming of Vp1236 as HexR is not supported sufficiently. As the authors note, HexR is a member of a family of related

proteins HexR/MurR/RpiR. HexR was first named in Pseudomonas aeruginosa in 1990 (https://doi.org/10.1128/jb.172.11.6396-6402.1990). Proctor later reported that P. aeruginosa HexR shared 56% identity with hypothetical regulatory protein in V. cholerae (https://www.proquest.com/dissertations-theses/genetic-biochemical-characterization-hexr/docview/304470780/se-2). This % identity is not reported in the manuscript, only the % identity of among Vibrios. In Pseudomonads, hexR is adjacent to genes zwf and pgl (https://doi.org/10.1111/1751-7915.13263). This is not explained either. The hexR locus of Vibrios contain neither zwf nor pgl, but instead panP and adh, now shown in new S5Fig. So, while vp1236 may encode a transcription factor that participates in controlling some gene similar to those controlled by P. aeruginosa HexR, the evidence presented here and prior do not support that Vp1236 is HexR. Again, functional complementation of a V. parahaemolyticus hexR with P. aeruginosa hexR, for example, would provide rigorous proof. Without such proof, HexR designation is speculative.

-The reviewer has indicated an important matter relating to HexR nomenclature, something that we gave considerable thought to as we prepared the initial manuscript and figures. We now include an improved and better rationale for naming Vp1236 as HexR in the revised manuscript, including percent identities and similarities (see lines 233-237). We also include citations for the original Pseudomonas literature in 1990. We have opted to not perform Pseudomonas genetic complementation in Vibrio based on the following rationale:

As pointed out, Vp1236 is a member of the HexR/MurR/RpiR family of transcriptional regulators. Our decision to name Vp1236 HexR is based on previous literature, specifically detailed studies by Leyn et al (2011) Journal Biological Chemistry, and by Campilongo et al (2017) in PLoS Genetics. Importantly, we cited these papers in the manuscript to support our interpretation of Vp1236 as HexR. As authors, we agree with the published HexR literature, and our view is that our data for Vp1236 (V. parahaemolyticus HexR) aligns very closely with the phenotypes and experimental observations for HexR in other proteobacteria, including *Pseudomonas*. While Genus level differences do exist for the transcriptional regulon members, a HexR overarching role in central carbon metabolism regulation is strongly maintained. In fact, we would be going against the published literature to name Vp1236 differently. Leyn et al 2011, previously identified Vp1236 as a HexR orthologue and the RegPrecise database provides a comprehensive view of HexR (Vp1236) regulon members (and for multiple other *Vibrio* species).

The reviewer is correct that *hexR* gene synteny (gene neighbours) differs between *Pseudomonas* and *Vibrio* species. Importantly, gene synteny is not always an accurate feature that predicts gene function. For example, gene or chromosomal re-arrangements can occur, resulting in altered genetic organization (gene location) while maintaining gene function. It is unclear why the gene synteny differs between Pseudomonas and Vibrio. Within the Vibrios, gene synteny appears to conserved and we provide a figure to indicate this feature.

We also looked at a predicted protein structure for Vp1236 (HexR) compared to Pseudomonas HexR (PA3184) using alphafold. The two structures appear remarkably alike and when overlaid show substantial overlap (see below):

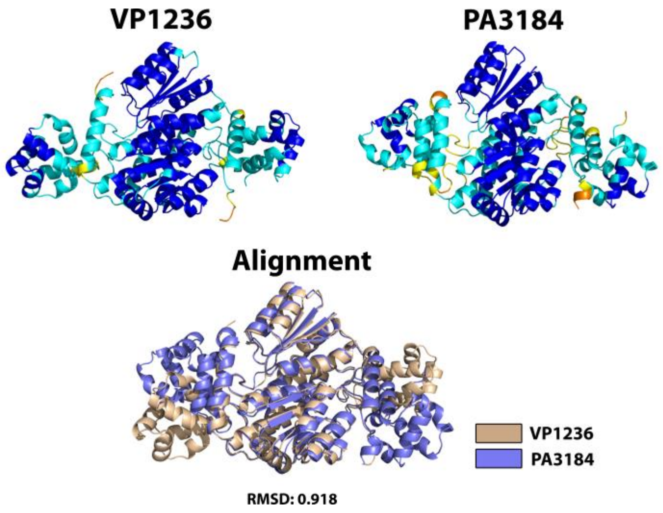

While this is not empirical data, it provides considerable support for interpreting Vp1236 as a HexR orthologue.

Another piece of supporting evidence can be found in sequence similarity scores. Vp1236 exhibits high sequence similarity to Pseudomonas HexR (59% identity, 77 similarity). Alternatively, if one takes the HexR sequence from *Pseudomonas aeruginosa* (PAO1) and performs a BLAST search against the *V. parahaemolyticus* genome, only a single protein is returned and that happens to be Vp1236.

Going beyond protein structure and sequence similarity, the phenotypes linked to HexR function are generally shared between *Pseudomonas* and *Vibrio*. The main theme is that HexR regulates central carbon metabolism and carbon flux. We demonstrate this aspect for Vp-HexR with empirical data for three very different carbon sources (peptides, glycerol, and chitin for the first time in any bacterial species). Moreover, we provide empirical data for two predicted HexR gene regulon members (*pgi* and *glgX*).

Overall, our analyses and empirical data provide multiple lines of evidence to support naming Vp1236 as HexR. Importantly, our data aligns with previously published literature pertaining to HexR in other bacteria and how others view this protein's function. In other words, we have not obtained any data or observations that would provide an explanation or justification to name Vp1236 anything else other than HexR.

2. New Fig S6. The GFP containing strains are not isogenic. To be rigorous, strains should carry the GFP marker in the same location. One would also expect the same reduction in growth of the mutant when the marker is swapped: WT is unmarked (GFP-), and the deletion mutant is marked (GFP+).
For additional rigor we have also done the experiment the reviewer has asked for GFP marker swapping. We found the same growth reduction for hexR deficiency in a competitive context (as expected). We present this as supplementary data (See S6 Fig)

-The reviewer's proposed approach for isogenic insertions is reasonable however the fact that none of the genes where transposon insertions occurred are functionally important for chitin metabolism based on Tn-seq data must be considered. The experiments with three different GFP gene insertion HexR deficient strains provide three independent observations, and each revealed a consistent and reproducible reduction in chitinolytic competitive growth compared to HexR expressing bacteria.

3. Fig 7B. This panel is qualitative and not interpretable as shown. It should simply be removed.

-As recommended, the panel has been removed and the text in the results has been deleted.

4. In response to Comment 14 by Reviewer 1, the authors state that "RegPrecise was initially used to identify putative HexR binding sites upstream of specific genes. For the revised manuscript, we selected two genes (pgi and glgX) with HexR binding sites that either scored highly to the consensus HexR binding site or were implicated from other studies." I recommend a supplemental table listing all the specific genes predicted by RegPrecise to have a putative binding site for Vp1236. Since pgi and glgX "scored highly", it would be appropriate to show the scores for each gene on the list as well to an explanation of how the genes were prioritized. It is also noted (lines 374-376) that there are no citations to other studies used for selecting pgi and glgX, in contrast to this comment in the rebuttal.

-This has been done as recommended and can be found in supplementary data file 4 and in the text at lines 387 onwards. As requested, an appropriate citation has been added on line 397.

Minor comments

1. New Fig. S7. Please provide the consensus sequence generated from RegPrecise here, as in the text (397-398) and indicate in the yellow highlighted sequence, the nucleotides of the predicted binding sites that match the conserved.

This has now been added.

2. New lines 242-248. The authors should acknowledge that by 36 h, the cell density of the uncomplemented deletion mutant exceeded the WT or complemented mutants, with no decline observed. Why might this be?

-We have edited the text to better convey the observations in the data (see lines 269 onwards).

One interpretation is that HexR negatively regulates some central carbon pathway genes while it also serves to positively regulate other genes. In the absence of HexR, the normal regulation is lost and likely pleiotropic leading to altered carbon flux and assimilation. The same could also be true for HexR overexpression, which is exactly what we observed in altered growth dynamics (compared to wild type or normal HexR levels).

If we went beyond 36 hours, we predict that the uncomplemented deletion strain would show reduced optical density in spent media. That being stated, the purpose of the experiment was to effectively demonstrate the role of HexR in carbon assimilation in a burdening spent media condition. The data indicates that HexR acts to coordinate early and efficient carbon assimilation.

In contrast, for HexR absence, the bacteria exhibit a considerable lag in growth but will eventually assimilate carbon and grow in a HexR-independent manner.

3. New line 265-267. The above point could be mentioned here as well.

-We agree and have modified the text accordingly (see lines 269 onwards)

4. The last sentence of the Abstract should read “support” rather than “supports”

-Corrected.

Reviewer #3: The authors did an excellent job responding to my comments and those of the other reviewers. It is quite impressive how well they addressed critical method issues and improved the clarity of the manuscript and conclusions. Well done.

-Thank you for the supportive comments.
