# Supplementary material for: Functional genomics of chitin degradation by Vibrio parahaemolyticus reveals finely integrated metabolic contributions to support environmental fitness
Source: PLoS Genet. 2025 Mar 3;21(3):e1011370. doi: 10.1371/journal.pgen.1011370 (PMC11906056; doi:10.1371/journal.pgen.1011370)
Supplement: Attachment — Submitted filename: Author_Response to Reviewer Comments.pdf [file pgen.1011370.s012.pdf]

Reviewer #1: In this work by Getz et al, the authors use a Tn-seq approach to identify genes that play a role in chitin-based growth. The question is timely and relates to an important open question in microbiology. The method is also appropriate and led to two main discoveries. The first discovery is that OprD is a possible novel chitin transporter. The second discovery is that HexR, a TF, is required for growth on chitin but also has more general effects on V. parahaemolyticus physiology. Overall, I found the work interesting and the discoveries quite novel and intriguing. My main concerns are that in many places, the conclusions are overinterpretations of the available results, and parts of the work would benefit from strengthening the supporting data.

Thank you for the interest and assessment of our manuscript. We have made changes based on your recommendations and those from the other reviewers. New experiments and analyses provide additional rigor and details. We hope you find the revised version extended and much improved.

Major comments:

1. Lines 172-3 (Fig. 4): If the statistics were performed on the different strains for each timepoint, such a claim (increase in cell numbers between 60 and 80 hrs) should be further supported by statistical analyses between timepoint within the same strain.

-We agree and have performed new growth curve experiments and include pertinent statistical analyses to support the stated conclusions (see new Supplementary Table S4) Please note that we have generated an additional chiP mutant strain and a chiP/vp0802 double mutant strain to strengthen the study and include the data here for a comprehensive and comparative assessment for each chitoporin contributions to chitinolytic growth. The double mutant has a severe growth defect when grown on colloidal chitin or large shrimp flakes. This data can be found in Figure 4B and within the main text on page 20, lines 445-447

2. Line 175 (Fig. 4): Without knowing the statistics of the data for the 24 hour timepoint, the complemented strain appears to have two growth phases (first 24 hours and last 24 hours). Can the author please comment on that? Also, the 60-80 hour phase is the same time frame in which the uncomplemented mutant shows growth, and the statistical analysis in the graph suggests that the difference between the strains is not significant. Can the authors please comment or explain this as well?
-We now include statements relating to growth phases with statistical analyses to quantitatively assess any differences in growth for the bacterial strains, including the new chiP and chiP/vp0802 mutant strains.

3. Line 179 (Fig. 4C): To claim outer membrane localization - did the authors check whether the outer membrane preparations were "pure" or contained the contents of other cellular compartments (e.g. by blotting for known periplasmic or inner membrane components)? Also, another band appears specifically in the mutant at ~25 kDa, and a band between 20-25 kDa is apparent in the WT and complemented strains but not the deletion strain. Can the authors comment on those? Have they tried to identify them?
The outer membrane preparations were described as ‘fractions’ and we did not present or

state that the preparations were “pure”. The outer membrane fractionation procedure is based on selectively solubilizing inner membrane with sodium lauryl sulfate. We have provided literature citations relating to this in the revised manuscript. To enrich outer membranes, we performed the exact same protocol that was used in the original ChiP discovery (V. furnissi) paper from the Roseman lab in 2000. After inner membrane solubilization (represented in gel below, lane 2), a high force ultracentrifugation step (150,000 x *g*) is used to sediment outer membrane (lane 3). Note the absence of the Vp0802 band in lane 2, and its presence in lanes 1 and 3. The supernatant after ultracentrifugation represents solubilized inner membrane components and cytoplasmic proteins (lane 2). The ultracentrifuge outer membrane pellet was ‘washed’ with fresh buffer and re-pelleted to thoroughly reduce residual inner membrane material.

We don’t have specific antibodies to inner or outer *V. parahaemolyticus* membrane proteins for blotting experiments. Instead, we show a protein gel that indicates various steps of the cell fractionation and outer membrane isolation procedure. It is evident, that each step has a different protein banding profiles which reveals the efficacy of detergent solubilization and differential ultracentrifugation towards an enriched outer membrane preparation.

Regarding the protein band at 20-25kDa in the outer membrane protein profile. We believe this is a degradation product. It was not repeatedly present in other preparations. It is also does not appear as a major species in the total cell lysate (after sonication, compare lane 1, lysate vs lane 3,outer membrane enrichment). At the current time, it is difficult to make any specific or accurate statements about the protein species which seems to be variable in its appearance in our sample preparations.

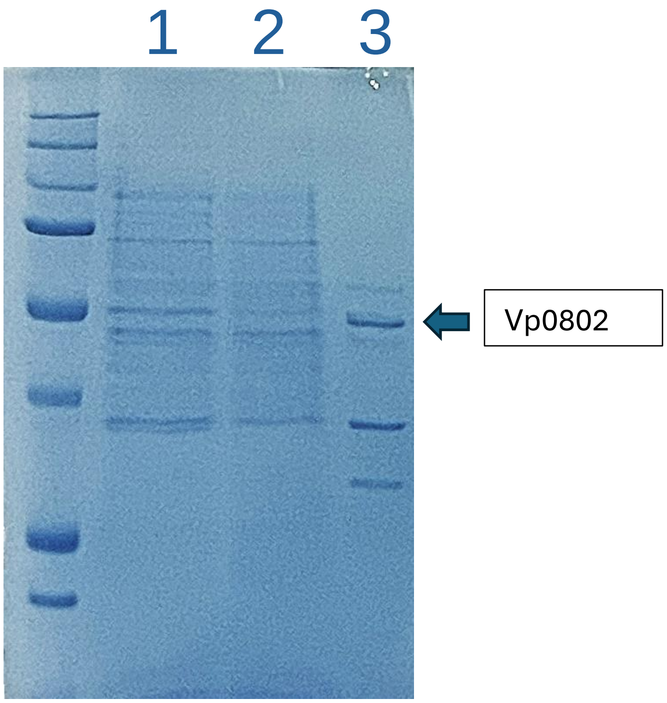

4. Fig. 5C-D: Can the authors please explain why the growth pattern of the complemented mutant is so different in 5C and 5D? In C, the OD decreases after 10 hours, whereas no decrease in OD is observed in D for up to 25 hours. Unless I misunderstood something, both panels show growth in spent media.

The reviewer is correct. The graph in 5D was performed with aerobic shaking conditions and is different from the experiment in 5C (static growth). We apologize for this error and now

show experiments with accurate descriptions. Importantly, we include the appropriate statistical analyses to support our stated conclusions. We thank the reviewer for finding the initial error. The new data (Figure 5C) is improved, much more coherent, and allows for confident interpretations.

5. Fig. 6B: If I understand the methods correctly, these GFP strains are actually random Tn5 -inserted mutants, and the authors do not know what is the additional mutation. If this is true, then the assay is quite "messy", and the results should be interpreted with caution (or the experiment should be performed on several independent Tn colonies to confirm that any observed phenotype is not due to the random Tn5 mutation).

We are in complete agreement. To address this matter, we performed genetic marker retrieval experiments on three independent hexR Tn5 transposant strains and a wild type strain transposant. This approach identifies the exact chromosomal insertion of the transposon. In each case, the respective strains were found to be disrupted in genes that do not contribute to growth on chitin, based on the empirical categorization derived from the Tn-seq and NGS datasets (Table S1, Fig S5). In other words, the Tn5 gene disruptions map to genetic loci that are non-essential for growth on chitin, and the respective strains do not have a general growth defect.

For additional rigor, we performed competition experiments with three independent hexR Tn5 strains (competing against wild type). The average competitive index was found to be 0.4 revealing a statistically significant competition defect for hexR loss function.

This data is now presented in Fig 6B and 6C and the genotype and description of the strains are included in Fig S5 (in addition to the strain list associated with the study, Table 1).

6. Fig. 7: These results suggest a negative effect of the hexR deletion on swarming. The authors then show a positive effect of the deletion on swimming. Why haven't the authors also quantified swarming mobility on agar to strengthen their claims?

We evaluated swarming as recommended. Based on our experiments using swarm agar plates, we found no apparent difference for wild type and the *hexR* mutant. We went further and generated a *lafk* promoter fusion to gfp to report on *lafK* gene expression. It was observed that swarmer cells near the periphery of growth were filamentous and expressing GFP. This was equally true for wild type and hexR mutants. Statements in the text can be found on page 16, lines 345 onwards. The new data is also presented Fig 9E.

Minor comments:

1. The first paragraph of the Results section (lines 79-85) should include an explanation of the selective chitin conditions. Only selection on glucose is currently mentioned before progressing to descriptions of DNA isolation.

The selection described in the first paragraph of the results is only for the generation and selection of the transposon mutants from the parent strain and does not describe the selection performed for essentiality analysis. The reviewer is correct that this was confusing, as we did not explain the following fitness bottleneck selections on glucose and on chitin. This has been amended in the text to better explain this procedure in the 2nd paragraph of the results, page 5, line 86-88.

2. Line 100: Can the authors Please explain how are 627 genes essential in both conditions if only 611 were essential for growth on chitin?

We apologize for this oversight and thank the reviewer for catching this error. We have re-

computed the number of essential genes for both conditions based on the HMM outputs and have corrected the number to 539. See page 15, line 103.

3. Lines 160-161: It would be nice to visually show the level of conservation and synteny of these homologs in other vibrios known (or not) to use chitin as a carbon source, to get a sense of the level of conservation of this seemingly important component.
Thank you for this recommendation. We have generated a schematic that nicely indicates conserved gene synteny (flanking/adjacent genes) for *V. parahaemolyticus hexR* orthologs in *V. cholerae* and *V. vulnificus* (see new Fig S4). The analysis reveals genes encoding i) an iron containing alcohol dehydrogenase (*adh*) and ii) amino acid decarboxylase (*panP*). The intergenic region between *panP* and *hexR* in each case is just over 300 base pairs, indicative of *hexR* being transcribed from its own upstream promoter. Consistent with this interpretation, a DNA amplicon including the upstream (intergenic) *hexR* region and *hexR* coding sequence restored HexR functionality to a hexR null mutant strain via trans-complementation.

4. Lines 164-6: Can the authors indicate whether the structure prediction was performed on the entire protein sequence containing the signal peptide or after its presumed cleavage? Also, perhaps showing the known or predicted structure of other experimentally investigated OprD family members will provide a better visual appreciation of the similarity of this protein to the rest of the family.

We have generated a predicted structures of the Vp0802 OprD-like porin without its signal sequence (Fig 4A) along with an overlapping structure image with *E. coli* OprD (Fig S1) to demonstrate high structural similarity for this protein family. Even with modest sequence conservation (29% identity, 43% similarity), this single channel porin family displays remarkable beta barrel structural conservation. We include models of ChiP trimers to clearly demonstrate that the single channel OprD-like porin and ChiP porin complex are indeed structurally distinct (See Fig S1).

5. Can the authors please add an explanation on how the OprD complementation plasmid prepared?
We now provide details in the methods for the vp0802 genetic complementation plasmid. The vp0802 gene is under the control of its own promoter and cloned into pVSV105, a stable *Vibrio* derived shuttle plasmid with chloramphenicol resistance selection. Please see page 28, line 609.

6. Line 178: Regarding the “Late stationary phase”, can the authors please clarify what timepoint was used to take cells from, since the growth curves are different for each strain.
As recommended, we have included more detail in the text (see page 9, line 173 onward). The reviewer is correct as the growth curves are indeed different.

7. Lines 206-7 (Fig. 5A): I think that all strains reach stationary phase at 400 min, since at this time point the angle of all the curves changes.
We agree and have modified the text accordingly (see page 11, line 222 onwards). Under these static growth conditions (96 well plate), there appears to be a second phase or a diauxic growth pattern. Notably, beyond 400 minutes, there is additional growth for the wild type and complemented strains whereas the hexR null mutant is significantly impaired. This key observation was the basis for the spent media experiments that followed.

8. Lines 227-230 (Fig. 5C): The growth (or rather lack thereof) is similar between the mutant and the wild-type strains. Why are the authors focusing on the mutant here? Also, the decrease in OD600 starting at ~600 min for the complemented strain is interesting. Can the authors comment on whether this can be cell lysis or perhaps aggregation? Have they tried to determine viability by CFU?
We thank the reviewer for this important recommendation. Indeed, the lack of growth for the wild type and mutant was consistently observed. The sharp decrease in OD was also challenging to address. We changed the experiment from static to shaking conditions (more aeration) and found that the all the strains could indeed grow under these nutrient limiting conditions. We still find that HexR overexpression supports an initial faster growth rate over wild type bacteria and the *hexR* deficient strain. Furthermore, we also observed reduced OD readings for HexR overexpression strains at late time points, suggestive of cell lysis. At later time points, wild type and the *hexR* mutant demonstrate growth after a considerable lag phase, the lag phase being longer in time for the *hexR* mutant. These new data highlight the critical role of HexR for contextual regulation of nutrient (carbon) assimilation. Additionally, we have modified the text to indicate that HexR overexpression supports dysregulated early growth under nutrient limiting conditions. See page 12, line 244 onwards for these changes.

9. Lines 237-9 (Fig. 5D): The data points in panel D are few and far apart. It is difficult to make the claims listed here based on the available results. For example, the author can say that the WT growth surpassed the other strains sometime between the 12h and 24h timepoints, but not that it happened "after 24 hours of growth"
We have removed Fig. 5D from the manuscript as the experiment was performed differently from the others in the same panel. We apologize for this oversight and now present a complete data set that allows for better comparisons.

10. Fig. 5D: I wonder whether the authors monitored viability (CFU) and not only OD, since OD readings can be misleading, especially after long incubation periods that may lead to the formation of biofilm or cell aggregates that could affect OD readings.
-As stated above for #9, 5D has been removed with an improved experiment using aerobic shaking conditions which supported growth for all bacterial strains allowing for better comparisons (See new Figure 5C in revised manuscript)
11. Lines 247-8 (Fig. 6A): Since there are no measurements between 4h and 24h, all the authors can say here is that there is a defect during the first 4 hours. However, since the initial OD of the mutant was lower than that of the WT, I am not sure that they can even claim that.

We agree that the early time point statement is not justified. We found that the bacteria consistently have an extended lag phase upon subculture into chitin media from overnight growth in Luria Broth Salt (LBS). This is expected given the stark change in carbon sources. What can be stated is that the growth rate for the *hexR* mutant (increase in cell number over time) is significantly higher than wild type between 24-30 hours. We have made changes in the main text to better convey that information. See page 13, line 270.

12. Fig. 7A: Please clarify which crystal violet image belongs to which strain.
For simplicity, we have decided to remove the crystal violet images and to rely on the quantitative spectrophotometric data for data presentation. The crystal violet images for the respective cuvettes were initially shown for completeness but are unneeded.

13. Line 286: Why are the data not shown?
We now show the data as required and to be consistent with PLoS Genetics policies.

14. Lines 314-6: Can the authors please elaborate on how the predicted promoters were identified?
RegPrecise was initially used to identify putative HexR binding sites upstream of specific genes. For the revised manuscript, we selected two genes (pgi and glgX) with HexR binding sites that either scored highly to the consensus HexR binding site or were implicated from other studies. In each case, the entire 5' upstream region (intergenic DNA sequence) was cloned into a plasmid harboring a promoter-less luxCDABE cassette. We included the entire intergenic region with the rationale that a HexR binding site and promoter would likely be found within that region of DNA. We now include a supplementary figure (Fig S7) that localizes putative promoter elements for the respective genes and HexR binding sites based on the consensus binding sequence.

15. Fig. 8: The conclusions would be strengthened if the authors can show the complementation of HexR downregulates these promoters.
We have performed this experiment, and the outcome is as expected. To do this complementation experiment, we had to use a plasmid that was compatible with pVSV-lux. This was achieved using pDSK519, a broad host range plasmid often used in gram negative bacteria. The *hexR* gene is cloned to be under transcriptional control of its own promoter. Critically, HexR that can act as a negative or positive regulator. We found that trans-complementation over-repressed or over-activated the reporter plasmid (more than wild type), presumably due to copy number effects (excess HexR expression). Complementation did not work well for the zwf gene reporter construct strains yielding inconsistent datasets. Correspondingly, we have decided to remove that construct and associated *zwf* statements from the revised version. The new data is shown in Figure 10.

16. Have the authors considered testing whether HexR affects OprD expression directly?
We tried oprD promoter-lux fusions in wild type and hexR mutant bacteria respectively. We did not see any major differences under chitinolytic growth although the experiment was complicated as accurately enumerating bacteria was challenging. Simple optical density readings were not reliable due to colloidal chitin particulates in the sampled cell culture required for measurements. To address this point, we comparatively evaluated outer membrane protein fractions by SDS-PAGE prepared from wild type and hexR mutant bacteria. The Vp0802 (OprD) protein at 50kDa was qualitatively equivalent for the strains, suggesting that HexR is not a major contributor to Vp0802 expression levels. Moreover, no evidence for a DNA sequence resembling the consensus HexR binding sequence was found within the promoter region of *vp0802*. We have not included this data in the revised version for brevity but can do so upon request.

17. Lines 375-7: Have the authors considered testing whether the hexR mutant is indeed deficient in its ability to bind chitin flakes?
Thank you for this suggestion. We generated a *hexR* mutant GFP tagged strain and cultured these bacteria on shrimp chitin flakes as a sole carbon source. Microsopy analyses revealed fluorescent green *hexR* mutant bacteria in close association with chitin flakes. The cells were primarily short rods. In contrast, wild type bacteria exhibited filamentous forms that were readily apparent (see new data in Figure 7B)(see page 15 line 312). Given the *hexR* mutant

strain ability to grow on colloidal chitin, the chitin association (binding) result is expected, perhaps being mediated by a pilus or cell surface adhesin. We don't have an established biofilm assay for *V. parahaemolyticus* on chitin flakes, so we have chosen to not make any statements in the main text about *specific* biofilm chitin flake interactions.

18. Line 409: I don't think that 85% sequence identity is "modest" conservation.
We agree. That being stated, Vp802 orthologs are present in many Vibrio species with varying levels of sequence identity. We present this data with a comprehensive list generated using reference genomes for a wide diversity of *Vibrio* species. See new data in Fig S1

Reviewer #2: Vibrio parahaemolyticus (Vp) is a marine bacterium that can cause gastritis when ingested. As a member of the marine community, like other Vibrios, it plays an important role in nutrient cycling by metabolizing chitinous material, the most abundant polymer in the marine environment. Despite chitin metabolism being well studied in Vibrios for decades, the authors here identify by Tn-seq two new regulators of chitin utilization in Vp. Also shown are effects of mutations in these two genes on growth and phenotypes such as biofilm formation. The manuscript is well written and the rationale described carefully.

Thank you for the kind and detailed assessment of our manuscript. We incorporate several changes based on your recommendations (and those of the other reviewers). We feel the revised version is extended and much improved based on the recommendations.

Major comments

Lines 201-203. What is the % identity of Vp1236 to Vibrio vulnificus (Vv) HexR? I was unable to find a publication of V. cholerae HexR. An alignment of the Vp1236 and Vv HexR protein sequences would be useful. Where are the amino acid differences? In a putative DNA binding domain (appropriate to show in an alignment)?The same gene name should only be used if the authors demonstrate functional complementation of a Vp hexR deletion mutant with Vv hexR. Or the reverse. An inability to complement could suggest Vp1236 is similar to HexR, but not actually HexR. Are vp1236 and Vv hexR syntenic? This would support the opinion that the two are homologs. A comparison of the organization of the genomic loci for both vp1236 and Vv hexR would also be useful.

We now include a schematic that demonstrates that the genomic locus around vp1236 (hexR) is highly conserved (syntenic) in *V. vulnificus* and *V. cholerae*, being flanked by *panP* (gene encoding glutamate decarboxylase) and *adh* (iron containing alcohol dehydrogenase) (See new Fig S4). The respective encoded HexR proteins are highly conserved with sequence identity scores of 95% (for *V. cholerae*) and 94% (for *V. vulnificus*). Given this very high level of sequence identity, the need to perform genetic complementation seems unnecessary to confirm relatedness or functionality.

What strains are referred to in lines 284-6? And the second half of the sentence is insufficient. What biofilm biosynthetic pathways are being referred to? There are more than one biofilm biosynthesis loci - syp and scv (https://doi.org/10.1128/msystems.01226-21). What "pathways"? There are also multiple signaling pathways that control biofilm formation. Quorum Sensing? c-di-GMP signaling? Also, can't loss of a repressor such as qsvR and/or opaR still lead to biofilm formation despite indicating a mutation in a biofilm pathway? The qualitative term "robust" is not defined. Biofilm results would be bolstered by measuring

transcription of the two biofilm biosynthesis loci (syp and scv) by RNA-seq to demonstrate that transcription is unchanged in a hexR deletion mutant. It would also be interesting to explore the coordinate genetic regulation of biofilm formation by this new regulator and those already described. Specifically, one could test whether OpaR and/or QsvR overexpression overrides the biofilm defects seen in the hexR deletion mutant.

We thank the reviewer for these suggestions. We have revised our statement to be accurate and to not overstate the data. We did investigate biofilm formation under rich media (LB) and nutrient limiting conditions (MM9, with casamino acids and glycerol) and found that the *hexR* mutant was deficient in biofilm formation only under <u>nutrient limiting conditions</u> as revealed using a common crystal violet assay (see new data in Figure 7). Critically, genetic complementation full restored (if not increased) biofilm formation thereby revealing a functional role for HexR in contributing to biofilm formation only under nutrient limiting conditions. The detailed regulatory deficiency remains unknown at the current time however in the future (for a separate study), we intend to perform RNA-seq using the *hexR* mutant, especially given its many altered phenotypes under nutrient limiting conditions and its critical role for bacterial fitness. We expect to find many cellular pathways to be altered due to HexR absence.

Lines 293-301. Designating the filamentous cells as swarmer cells is speculative here. One could measure expression of lafK as in [21] as a criterion of swarmer cell differentiation. Without swarmer cell confirmation, the term “swarmer” should not be used in subsequent lines 298 and 301.

Thank you for this recommendation. Swarmer cell assays required special conditions which included brain heart infusion medium, dipyridyl (as an iron chelator), and high levels of calcium chloride. This is considered a rich medium with numerous carbon sources. From our multiple observations, HexR related phenotypes are less apparent under nutrient replete (rich) conditions. In nutrient limiting conditions (colloidal chitin growth, minimal medium), we consistently observed HexR dependent phenotypes, including cells that were significantly reduced for cell length (as determined for minimal medium and colloidal chitin, see new Figure 8 C and E).

Relating to lafK and swarming, we are excited to provide data for *lafK* promoter activity specifically in swarmer cells. We quantitatively measured *lafK* promoter activity using a *lafK* promoter-gfp transcriptional fusion. The data indicate *lafK* expression in cells sampled from ‘flares’ at the periphery of swarmer agar plate growth (see new Fig 9) for both wild type and the *hexR* mutant. Furthermore, we provide microscopy data that convincing demonstrates that wild type and *hexR* mutant bacteria can both surface swarm on agar. Moreover, the respective swarmer cells are elongated with *lafK* promoter activity consistent with a swarmer cell gene expression condition. We did observe significantly reduced *lafK* promoter activity for the *hexR* mutant compared to wild type bacteria, although it is unclear if this is biologically relevant.

In summary, *hexR* mutants appear impaired for filamentation under nutrient limiting conditions. We have removed the term ‘swarmer cell’ for this specific context. Under nutrient replete (rich) conditions that promote swarming, *hexR* mutants do elongate and form swarmer cells via lafK expression for lateral flagella production. We thank the reviewer for recommending this line of investigation as it added HexR context and more rigorous experimentation.

Figure 7. It would be interesting to know whether biofilm formation occurs with chitin as a sole carbon source and what cell morphologies (rod shaped, curved) are observed in the periphery and center of colonies on LB. More details regarding cell lengths would be useful as well.

We have made measurements of cell lengths and provide this data as a new Figure 8D. We chose to focus on MM9 growth conditions (nutrient limiting) where we observed significant cell length differences for the strains. We have also evaluated cell morphologies on swarmer plates (rich media), specifically at the periphery of growth near 'flares' (see Fig 9).
We are not readily equipped to perform proper chitin biofilm experiments. To address this point, we cultured GFP tagged bacteria on shrimp flakes as a sole carbon source and imaged the flake material using DIC and fluorescent microscopy. While we can't make any biofilm specific statements, we did observe that wild type bacteria had a mix of curved and filamentous cells, whereas the hexR mutant was mostly curved rod shaped (see Fig 7B). We thank the reviewer for recommending these experiments as they provide a better understanding of cell morphologies that are impacted by HexR under a variety of growth conditions.

Minor comments

Line 152. Replace "cell" with "periplasm"
This change has been made within the text.

Line 161-2. Citations needed? What are the % AA Similarity and % AA Identity with regard to the other Vibrios?
We include a citation as recommended. A new figure (Fig S1) now shows a comprehensive list of Vibrio species with a heat map for percent amino acid sequence identity.

Lines 166-168. The SignalP analysis is consistent with its export beyond the cytoplasm into the periplasmic space. But provides no information supporting transport or localization to the outer membrane.
We have incorporated the change, 'consistent with protein export beyond the cytoplasm'.

Line 171. Since no results are shown that determine an alternative chitin growth pathway, I suggest the authors qualify this statement "…can presumably utilize…"
We now include data and observations for a vp0802, chiP double mutant in the revised manuscript. The data revealed that the double mutant was severely compromised for growth when colloidal chitin was the exclusive carbon source in the medium.

Line 220. Please provide a more detailed explanation of the complementation here. Plasmid-borne? Approximate copy #? Under control of what promoter?
A detailed description is now provided. In all cases, we use gene specific promoters in our genetic complementation constructs. This has been updated in the methods and a description of the plasmids is included in Table 1.

Line 227. Were the WT cells grown to late stationary phase in LB or MM? With glycerol? With chitin?
The wild type cells were grown to late stationary phase in LB. This has been added to the main text for clarity.

Line 292. with chitin? I assume with glycerol based on line 213, but should be clear here too or create an abbreviation for example: "MM9+glyerol" vs "MM9+chitin"
We have inserted MM9+ glycerol. Thank you for this recommendation as it improves clarity and readability of the main text. We have also included better descriptions for other growth conditions (experiments) as well.

Line 328-329. does location of putative HexR binding site support its role as a DIRECT repressor? For example, is there a putative promoter identifiable? Is the putative binding site near/with the -35/-10?
This is an excellent recommendation. We have mapped the predicted HexR binding site based on the consensus sequence as determined by RegPrecise. Indeed, we find that for vpa1645 (*glgX*), and vp2731 (*pgi*), a putative HexR binding site is present near -35/-10 promoter elements located upstream from the respective genes. We present these analyses in a new supplementary figure (See Fig S7)

Fig. 4. Legend has a typo. “b-barrell” should read “β-barrel (beta-barrel)”
This has been edited.

Fig 4. What might be the nature of the band at 23-25 kDa that changes in location with the different genetic backgrounds? This was not mentioned.
This point was also raised by another reviewer. The protein band at 20-25kDa in the outer membrane protein profile is likely a degradation product. It was not repeatedly present in other preparations. It is also does not appear as a major species in the total cell lysate (after sonication. At the current time, it is difficult to make any specific or accurate statements about the protein species which seems to be variable in its appearance in our sample preparations.

Fig. 7A. The images shown are of poor quality. I do not know how to interpret these. The images in Panel B could be enlarged and/or an inset used to more clearly show the morphology of individual filamentous and rod-shaped cells. Currently the images are too small to differentiate by eye clearly.
We apologize for the original images and have improved microscopy image quality within the revised manuscript. We include insets (larger images) to emphasize details as required.

Reviewer #3: This is a clearly written and ecologically relevant paper studying the genes required for growth of V. parahaemolyticus on chitin compared to glucose. The authors use classic Tn-seq experiments to delineate between growth on these two media. They identify expected genes and novel genes, and they chose a few to follow up. My major comment is regarding the organization of the manuscript. There seem to be four important findings (though the authors focus on three): Tn-seq confirms previously known or predicted chitin-growth required genes, T2SS genes, OprD-like porin, and HexR. The authors only briefly describe T2SS and don’t mention it in the abstract or discussion, so it seems like a minor finding in the context of the paper. The majority of the study focuses on HexR. In my opinion, this manuscript could be split into two; keep the current expanded study on HexR, but remove the T2SS and OprD findings, and follow up on OprD in another manuscript. The findings that ChiP is not essential in growth on chitin and that deletion of OprD leads to an increase in ChiP could be another story entirely. Alternatively, the deeper competitive fitness studies of the HexR could be moved to another manuscript and looked at further, leaving this study to

broadly focus on the Tn-seq and follow up on three systems: T2SS, OprD, HexR.

We thank the reviewer for these suggestions. We've opted to further develop the OprD-like porin data with additional mutant analyses. Moreover, we add new data related to HexR. Therefore, the revised manuscript retains two major foci but now with increased detail and depth for the OprD-like porin, and HexR functionality. Both foci are based on bacterial fitness during chitinolytic growth, as revealed by Tn-seq discovery.

Major comments:

• Fig. 4B: I'm not convinced that the data show restoration of growth in the complemented strain. Have the authors looked beyond 80 hrs? The increase in growth is so subtle, and one would expect a complemented strain to grow much more similarly to wild-type. This complementation was on a plasmid, perhaps too high copy (seen in Fig. 4C) is also detrimental to growth? I would suggest a chromosomal complementation.

We agree that the genetic complementation is delayed. It is performed in trans via a VSV105 plasmid with a natural Vibrio origin of replication (from pES213). Based on the suggestion, we extended our observations beyond 80 hrs and we were thus able to capture a better representation of the growth trends over time. The increase in growth is statistically significant, and we found the complemented strain to display a similar growth pattern (albeit at delayed time points) to the wild type strain (see new Fig 4B). We now include statistical analyses to support our statements and an additional table with statistics between time points (Data Table S3). Therefore, the plasmid supports chitinolytic growth albeit with different kinetics. We do note that the experiment is challenging as we seem to enter a viable but not culturable condition (VBNC), indicated by a sharp decline in viable enumerated cell counts at late time points (beyond 48 hours). <u>We did not observe cell lysis under these conditions</u>. Our interpretation is that under these sole colloidal chitin carbon source conditions, the preferred GlcNAc moieties derived from secreted chitinases have been rapidly consumed to support efficient growth and at later time points the cells must wait for chitin processing to catch up. This interpretation is consistent with a second inflection (increase) of cell counts or perhaps an exit from a presumed VBNC state.

In addition, we now report data for a vp0802, chiP double mutant that is severely impaired for chitinolytic growth (See Fig 4B). We show that a plasmid expressing either *vp0802* or *chiP* can partially restore growth to this impaired double mutant strain (Fig S3). These observations collectively demonstrate that our gene complementation is functional. The VSV105 plasmid is a derivative of a natural Vibrio fischeri plasmid (vibrio origin of replication) and is the most stable plasmid we have observed for genetic complementation studies.

• Fig. 4B: Are the colonies that eventually grow in the deletion strain suppressors? If re-inoculated, do these now behave as wild-type or closer to it?
We have investigated this possibility by carrying out subculturing experiments. We found that the deletion strain repeatedly showed a delay in growth relative to wild type. We can't rule out the possibility of genetic suppressors. That being stated, the increased expression and outer membrane localization of ChiP in the absence of Vp0802 is a phenotype that is completely consistent with chitin utilization. Moreover, we have since generated a *vp0802, chiP* double mutant which does not grow on colloidal chitin or shrimp chitin flakes. These data are

consistent with the view that Vp0802 and ChiP are the two main chitoporins that support the uptake of large chitooligosaccharides.

• The competitive advantage of the hexR- strain compared to wild-type in mixed cultures is really interesting. I would suggest that the authors test different ratios of mixing to see at what minimum proportion of the total the wild-type can overtake the hexR-. It is difficult to determine the full landscape of competition using only one ratio.

This might be a misinterpretation. The *hexR* mutant is actually competitively disadvantaged to wild type in mixed cultures (see Fig 6). That being stated, we did investigate altering HexR levels by overexpression in pure nutrient limiting culture conditions (not mixed). Here, overexpression of HexR in wild type bacteria or the *hexR* null mutant resulted in faster dysregulated growth compared to bacteria with normal levels of HexR (native wild type strain) (see Fig 5C). These data reveal that contextual HexR expression impacts growth dynamics, consistent with this regulator being involved in controlling over 20 genes involved in central carbon assimilation, including pgi, and *glgX* (see Fig 10). It is further complicated depending on the gene target; HexR can either be a negative or positive regulator, therefore HexR coordinates a multifaceted cytoplasmic carbon flux system and HexR effects would be expected to be carbon source and carbon availability dependent. Consistent with this view, we cite the report of Camilli and co-workers who found that HexR is an important fitness determinant in nutrient limiting conditions (pond water) for *V. cholerae* but not when nutrients and carbon sources are abundant (within host).

We agree with the reviewer on the interesting observation of bacteria lacking HexR demonstrating a faster growth rate compared to wild type bacteria under chitinolytic conditions (see Fig 6A). This is consistent with an interpretation of genetic dysregulation in the absence of HexR, with some metabolic pathways being upregulated. It is important to note that wild type bacteria demonstrate coordinated HexR dependent gene expression and growth under chitinolytic conditions. In mixed culture (wild type vs hexR null), the wild type outcompetes the mutant presumably due to coordinated gene expression mediated by HexR. These data collectively suggest that HexR is a critical fitness determinant during competitive chitinolytic growth, consistent with our Tn-seq observations.

• The finding that deletion of the OprD-like gene leads to an increase in ChiP is really important and seems understudied here. Does deletion of ChiP impact Vp0802? Are there differences in ChiP expression at different stages of growth?

To address this point, we have generated a *vp0802, chiP* double mutant. This mutant was severely impaired for growth when colloidal chitin was the sole carbon source. Genetic complementation with a plasmid expressing *vp0802*, or a plasmid expressing *chiP* partially restored growth to the *vp0802*, *chiP* double mutant (see Fig 4B and Fig S3). Therefore, both chitoporins are functional and can support the uptake of large chitooligosaccharides into the cell.

Notably, for the Vp0802 chitoporin, it appears to be dominantly expressed under liquid colloidal chitin conditions, with minimal ChiP expression (see Fig 4C). The mechanism behind this remains unknown and will be the subject of future studies. We haven’t explored

differential chitoporin expression for ChiP specifically. That being stated we present some comments in the discussion relating to contextual or niche specific expression for ChiP.

Minor comments:

• Lines 42, 59: Vibrio-mediated

Corrected, thank you

• Line 89: it would help to briefly describe the “chitin condition” here (even though in the methods already) – what is the difference compared to minimal medium with glucose? Just addition of chitin? Is it MM9+0.4% chitin? (line 246)

We agree and have made the change in the text for clarity.

• Reword sentence starting at 117: “Interestingly, there were several genes not conditionally essential in the chitin condition:” (then list).

We agree and have made this sentence structure edit.

• Line 129: Why not include vp1236 in Fig. 2?

Vp1236 is now included in Fig 2.

• Line 128: Has a previous publication shown that N-acetylated compounds are not transported in OprD porins? This struck me as lack of positive data in other studies, not a known specificity for these proteins. Perhaps reword to eliminate the latter part of the sentence. I also think the reference for the E. coli OprD-like protein that imports chitohexaose would be important to mention in the results section at the same part of the paragraph.

To address this point, we provide additional citations in the main text that have reported on substrate specificity for the OprD porins. It has been established that -COOH compounds are preferred substrates and that the inner porin channel has a ‘basic’ ladder of arginine and lysine residues that are hypothesized to interact with a substrate carboxyl group. In our initial submission, we did cite a paper reporting that N-acetyl cysteine, a compound with an acetyl group in addition to a carboxylate group, can interact with an OprD channel. Nonetheless, chitooligosaccharides do not contain carboxylate groups, therefore the Vp0802 chitoporin (OprD-like) specificity for these sugars is likely driven by interactions within the inner porin channel. In the case of the prototypic ChiP chitoporin, it is known that the C2 acetyl group of the GlcNAc is a specificity factor.

In the revised manuscript, we have included the above information and relevant citations, including the E. coli OprD-like porin in the results section as recommended
